# Genome-wide liver transcriptomic profiling of a malaria mouse model reveals disturbed immune and metabolic responses

**DOI:** 10.1186/s13071-023-05672-w

**Published:** 2023-01-30

**Authors:** Xueyan Hu, Jie Zhao, Junhui Zhao, Ence Yang, Mozhi Jia

**Affiliations:** 1 Department of Medical Bioinformatics, School of Basic Medical Sciences, Peking University Health Science Center, Beijing, 100191 China; 2 Department of Microbiology & Infectious Disease Center, School of Basic Medical Sciences, Peking University Health Science Center, Beijing, 100191 China; 3 Department of Physiology and Pathophysiology, School of Basic Medical Sciences, Peking University Health Science Center, Beijing, 100191 China

**Keywords:** *Plasmodium yoelii*, Blood stage infection, Cotranscriptome, lncRNA, circRNA

## Abstract

**Background:**

The liver is responsible for a range of functions in vertebrates, such as metabolism and immunity. In malaria, the liver plays a crucial role in the interaction between the parasite and host. Although malarial hepatitis is a common clinical complication of severe malaria, other malaria-related liver changes have been overlooked during the blood stage of the parasite life-cycle, in contrast to the many studies that have focused on parasite invasion of and replication in the liver during the hepatic stage of the parasite.

**Methods:**

A rodent model of malaria was established using* Plasmodium yoelii* strain 17XL, a lethal strain of rodent malaria, for liver transcriptomic profiling.

**Results:**

Differentially expressed messenger RNAs were associated with innate and adaptive immune responses, while differentially expressed long noncoding RNAs were enriched in the regulation of metabolism-related pathways, such as lipid metabolism. The coexpression network showed that host genes were related to cellular transport and tissue remodeling. Hub gene analysis of *P. yoelii* indicated that ubiquitination genes that were coexpressed with the host were evolutionarily conserved.

**Conclusions:**

Our analysis yielded evidence of activated immune responses, aberrant metabolic processes and tissue remodeling changes in the livers of mice with malaria during the blood stage of the parasite, which provided a systematic outline of liver responses during *Plasmodium* infection.

**Graphical Abstract:**

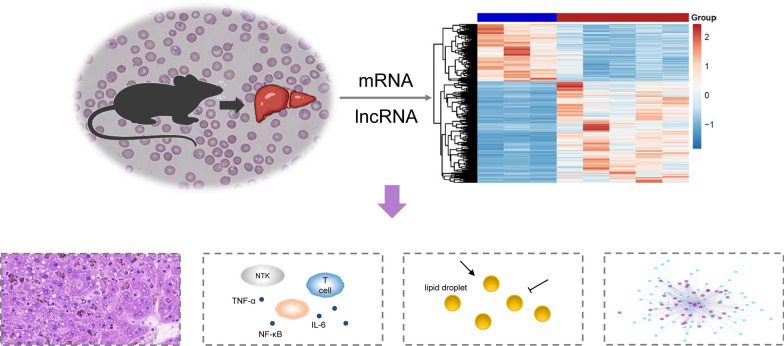

**Supplementary Information:**

The online version contains supplementary material available at 10.1186/s13071-023-05672-w.

## Background

Malaria is a widespread infectious disease that is prevalent mainly in tropical and subtropical regions of the world where it is a major health burden [1]. Systemic changes occur in organs of the infected organism, such as severe anemia and impaired microvascular perfusion, which are central to the development of severe malaria [2–4]. Severe malaria in turn is associated with ischemia/hypoxia, metabolic disturbances, hyperlactatemia and disturbance of consciousness, leading to high mortality [5–7]. Investigating the involvement and responses of important organs responsible for metabolism and immunity, such as the liver, may contribute to a systematic understanding of the pathogenesis of severe malaria and provide new perspectives on the treatment and care of critically ill patients [8, 9].

The liver is an important organ for maintaining immune and metabolic homeostasis. As part of the immune response, the liver plays a critical role not only in host defense but also in liver injury and repair [10]. During the liver stage of plasmodial infection, bone marrow-derived cells in the liver interact with the parasite, modulating the replication boost and the differentiation of sporozoites into merozoites [11–13]. During the blood stage of the infection, the liver is a main target organ for *Plasmodium*, as demonstrated by the activated immune responses and various liver injuries associated with high parasite loads observed in several malaria mouse models [14–17]. On the other hand, as a central metabolic organ, the liver integrates systemic metabolism to maintain glucose and fatty acid homeostasis. Impaired hepatic gluconeogenesis and hepatic lactate clearance have been reported to directly affect blood lactate concentrations, resulting in death-related acidosis in patients with malaria [6]. A positive association between high *Plasmodium*-infected erythrocyte (pRBC) load of the liver and jaundice, hepatomegaly and elevated liver enzymes has been observed in different malaria patient cohorts [18, 19]. Liver dysfunction in these patients is related to other organ dysfunctions and poor outcomes [19, 20], suggesting a potential role of the liver in malaria responses.

Compared with the liver stage of the parasite life-cycle, involvement of the liver in the blood stage of the parasite has been overlooked in most studies, and only a few animal and human studies on hepatic pathologies in malaria have been reported [14–16, 21, 22]. Histopathological evidence of the involvement of reactive Kupffer cells, retention of heme pigment, minimal pRBC sequestration and inflammatory responses in the pathogenesis of malaria-induced liver injury has been accumulating [15, 18, 23, 24]. However, systemic identification of molecular events is required to comprehensively demonstrate liver changes and provide a basis for exploring the supportive features of the liver response during infection.

In the study reported here, we explored the global transcriptomic changes in the host liver in *Plasmodium yoelii*-infected mice. We found that differentially expressed (DE) messenger RNAs (mRNAs) in the liver were mainly responsible for innate and adaptive immune responses. In contrast, DE long noncoding RNAs (lncRNAs) mainly focused on the regulation of fatty acid metabolism and oxidation‒reduction processes. Host genes highly coexpressed with *P. yoelii* genes were enriched in the processes of cellular transport and tissue remodeling. Additionally, hub genes of *P. yoelii* in the coexpression network suggested that conserved ubiquitination genes were highly coexpressed with host genes. Collectively, our work reveals that immune responses, metabolic changes and tissue repair are major events that occur in the liver during the blood stage of plasmodial infection and further provides potentially distinct regulatory roles of mRNAs and lncRNAs in liver responses.

## Methods

### Ethics statement

The International Guiding Principles for Biomedical Research Involving Animals were strictly followed during animal experiments. Procedures involving vertebrate animals were reviewed and approved by the Animal Care and Use Committee of Peking University Health Science Center with permit number PUIRB-LA2022677.

### Establishment of the* P. yoelii*-mouse model

Four-week-old male ICR/JCL mice (20–25 g) were obtained from the Department of Laboratory Animal Science of Peking University (Beijing, China; permit: SCXK-2012–0015) and housed under specific pathogen-free conditions. Mouse blood containing *P. yoelii* lethal strain 17XL (70% parasitemia) frozen in liquid nitrogen was thawed quickly for injection. Five mice were infected with 2 × 10^6^ infected red blood cells (RBCs) by the intraperitoneal route, similar to earlier studies [25, 26]. The parasitemia of infected mice was assessed by microscopic examination of Giemsa-stained blood smears post-infection. Five fields were randomly selected from each blood smear for cell counting, and the parasitemia percentage was measured as parasitized RBCs/total RBCs × 100.

### RNA extraction and transcriptomic analysis

As oxidative stress and hepatic apoptosis peak at 50%–60% parasitemia [26], mouse liver tissues were sampled at approximately 50% parasitemia. Total RNA was extracted from liver tissues using TRIzol reagent. The RNA integrity number (RIN) was assessed with an Agilent 2100 Bioanalyzer system (Aglient Technologies, Santa Clara, CA, USA), and extracted RNA with an RIN > 7.0 was retained for sequencing. Ribosomal RNA depletion libraries were prepared and sequenced with IGENECODE (Beijing, China) on the Illumina HiSeq PE150 platform (Illumina, Inc., San Diego, CA, USA).

### Read mapping and normalization of RNA sequencing data

RNA sequencing (RNA-seq) data were mapped to the reference genome of *Mus musculus* (GRCm39) using Hisat2 [27]. Gene expression levels were calculated with Stringtie [28]. Genes of *M. musculus* with average counts > 50 were kept for differential expression analysis. Circular RNAs (circRNAs) were identified with CIRI2 using the same RNA-seq reference genome for guidance [29].

### Identification and functional enrichment analysis

The R package DEseq2 [30] was used to identify DE RNAs. Genes with adjusted *P* values < 0.05 and fold changes > 2 were considered to be DE. Gene ontology (GO) and Kyoto Encyclopedia of Genes and Genomes (KEGG) pathway analyses of DE genes were carried out with DAVID v6.8 [31].

### Gene coexpression network construction and visualization

An artificial genome was generated by combining the reference genomes of *M. musculus* (GRCm39) and *P. yoelii* (GCF_900002385.2) [32]. The same RNA-seq data were mapped to the artificial genome as described above. *Mus musculus* genes with an average TPM (transcript per million) > 1 were kept, as were *Plasmodium yoelii* genes that were not expressed in the control group and had an average TPM > 1 in the infected group. The R package WGCNA [33] was used for gene coexpression network construction. Briefly, filtered and normalized expression data were used for average linkage hierarchical clustering. Network modules were identified using a dynamic tree cut algorithm with a minimum cluster size of 30. The correlation between modules and phenotypes (control and infection) was calculated, and the modules containing *P. yoelii* genes with a correlation coefficient > 0.8 were selected for downstream GO enrichment analysis. The visualization of special modules was performed with Cytoscape_v3.8.2 [34].

### Homolog identification among *Plasmodium* spp.

Reference protein sequences of *P. yoelii* were compared with those of a number of other* Plasmodium* species, including *P. falciparum*, *P. malariae*, *P. ovale*, *P. vivax*, *P. knowlesi*, *P. berghei* and *P. chabaudi*, using BLAST 2.10.1 + with a parameter e value = 1e10. Only proteins with > 90% overlapping sequences with other proteins and with overlapping sequences accounting for > 90% of the two protein sequences were considered to be homologous proteins.

### Hematoxylin–eosin staining of liver

Liver hematoxylin–eosin (HE) staining of infected and control mice was performed by Wuhan Servicebio Technology Co., Ltd. In brief, paraffin-embedded sections were deparaffinized and rehydrated using ethanol and xylene. HE staining was performed to evaluate inflamed cell infiltration and hepatocellular edema/necrosis/fibrosis.

### Real-time quantitative PCR

A total of 1 μg RNA was reverse transcribed into complementary DNA (cDNA) using the HiScript III 1st Strand cDNA Synthesis Kit (+ gDNA wiper) (Vazyme, Nanjing, China) according to the manufacturer's instructions. Then, real-time quantitative PCR (RT‒qPCR) analysis was performed using the Hieff UNICON® Universal Blue qPCR SYBR Green Master Mix (Yeasen Biotechnology Co., Ltd., Shanghai, China) according to the manufacturer's instructions. The cycling program consisted of an initial incubation at 95 °C for 2 min, followed by 40 cycles of 10 s at 95 °C and 30 s at 60 °C. Each gene was analyzed in biological triplicates and technical triplicates with the glyceraldehyde 3-phosphate dehydrogenase gene (*GAPDH*) as the internal reference. All primers used in RT‒qPCR assays are listed in Additional file 1: Table S1).

## Results

### Differential expression profiling of whole transcriptomes between infected and control groups

**Fig. 1:**
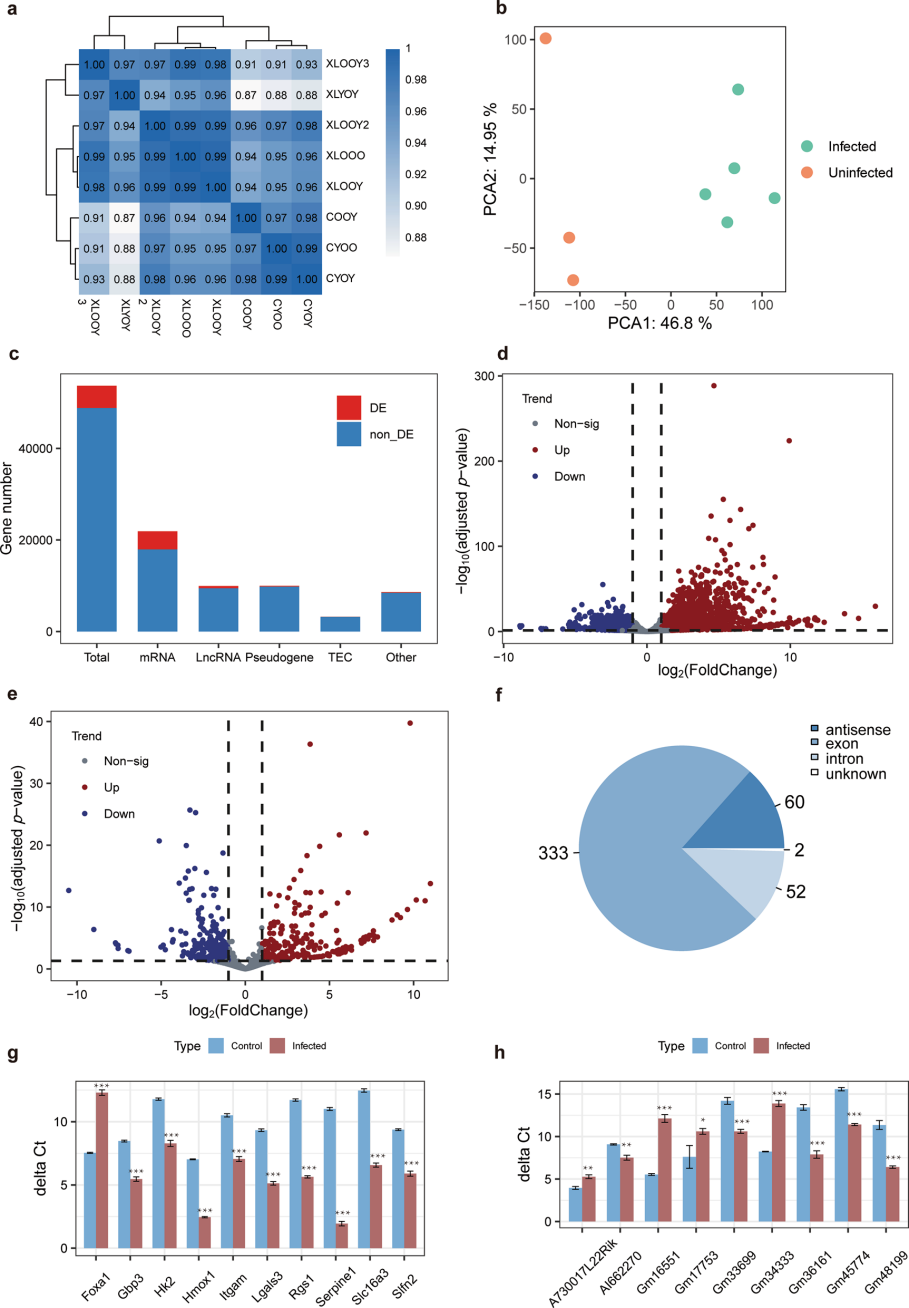
Transcriptomic overview of the *Plasmodium yoelii*–rodent system. **a** Pearson correlation and hierarchical cluster analysis of samples between infected groups and control groups, with five replicates for the infected groups and triplicates for the control groups. **b** Principal component analysis between two conditions. **c** Distribution of differentially expressed genes. **d** Volcano plot of differentially expressed mRNAs. **e** Volcano plot of differentially expressed lncRNAs. **f** Number and classification of identified circular RNAs in all samples. In the volcano plots, red and blue dots correspond to differentially expressed genes that are significantly upregulated or downregulated between the two groups (adjusted *P* value < 0.05, |log_2_FoldChange|> 1). The* X*-axis shows the log_2_(fold change) of expression, and the* Y*-axis shows the -log_10_(adjusted *P* value) for each gene. **g**, **h** Reverse transcription‒quantitative PCR of the top 10 differentially expressed mRNAs (**g**) and lncRNAs (**h**). The* X*-axis represents gene names, and the* Y*-axis represents the ΔCt value (Ct_target_ – Ct_endo_), with *GAPDH* as the reference gene. Boxes and error bars represent the mean ± standard deviation (SD) over triplicate biological samples and triplicate technical replicates of Ct values. The Wilcoxon rank sum test was used for ΔCt comparison. Asterisks indicate a significant difference at *P < 0.05, **P value < 0.01 and ***P value < 0.001. Ct, Cycle threshold; DE, differentially expressed; GAPDH, glyceraldehyde 3-phosphate dehydrogenase ; PCA, principle component analysis; LncRNA, long, noncoding RNA; mRNA, messenger RNA; TEC, protein tyrosine kinase Tec;  Non-sig, not significant

The *P. yoelii*-rodent model described in section Establishment of the *P. yoelii*-mouse model was established with noninoculated mice as the control group. Giemsa-stained blood smears of all five infected mice were examined until parasitemia reached 50% (Additional file 2: Figure S1). Liver tissues were resected from five infected mice and three controls for histological examination and RNA extraction. Hepatic sinusoidal expansion, hepatic cell edema, irregular arrangement of hepatic cords, isolated lipid droplets and the presence of macrophages engulfing a large number of malaria pigment particles were observed in the HE-stained liver sections of infected mice (Additional file 3: Figure S2). Total RNA was extracted from liver tissues for ribosomal RNA (rRNA) depletion high-throughput sequencing, and an average of 7.6 Gb of data were generated from each sample (Additional file 4: Table S2). RNA-seq of infected and control samples showed a high correlation within and between groups, indicating favorable data quality (Fig. 1a). Principal component analysis (PCA) indicated that biological replicates clustered together, while strong separation among infected and control samples distinguished the transcriptomes of the two groups (Fig. 1b).

To further explore the differences in the transcriptomes between the infected and control groups, we identified 4875 DE genes among 19,066 stably expressed genes using DEseq2 with thresholds of fold change > 2 and adjusted *P* value < 0.05. A total of 3975 mRNAs and 493 lncRNAs were found to be DE, accounting for > 90% of DE genes (Fig. 1c). In the infected group compared with the control group, 2537 mRNAs and 233 lncRNAs were upregulated and 1438 mRNAs and 260 lncRNAs were downregulated (Fig. 1d, e). We also identified 477 circRNAs among samples using CIRI2 (Fig. 1f), but no marked change in expression was found due to the low number of overlapping circRNAs of samples without RNase R enrichment. The top 10 DE mRNAs and lncRNAs were validated by RT‒qPCR (Fig. 1g, h).

### GO term and KEGG pathway analysis of DE mRNAs underscored the complexity of immune responses

**Fig. 2:**
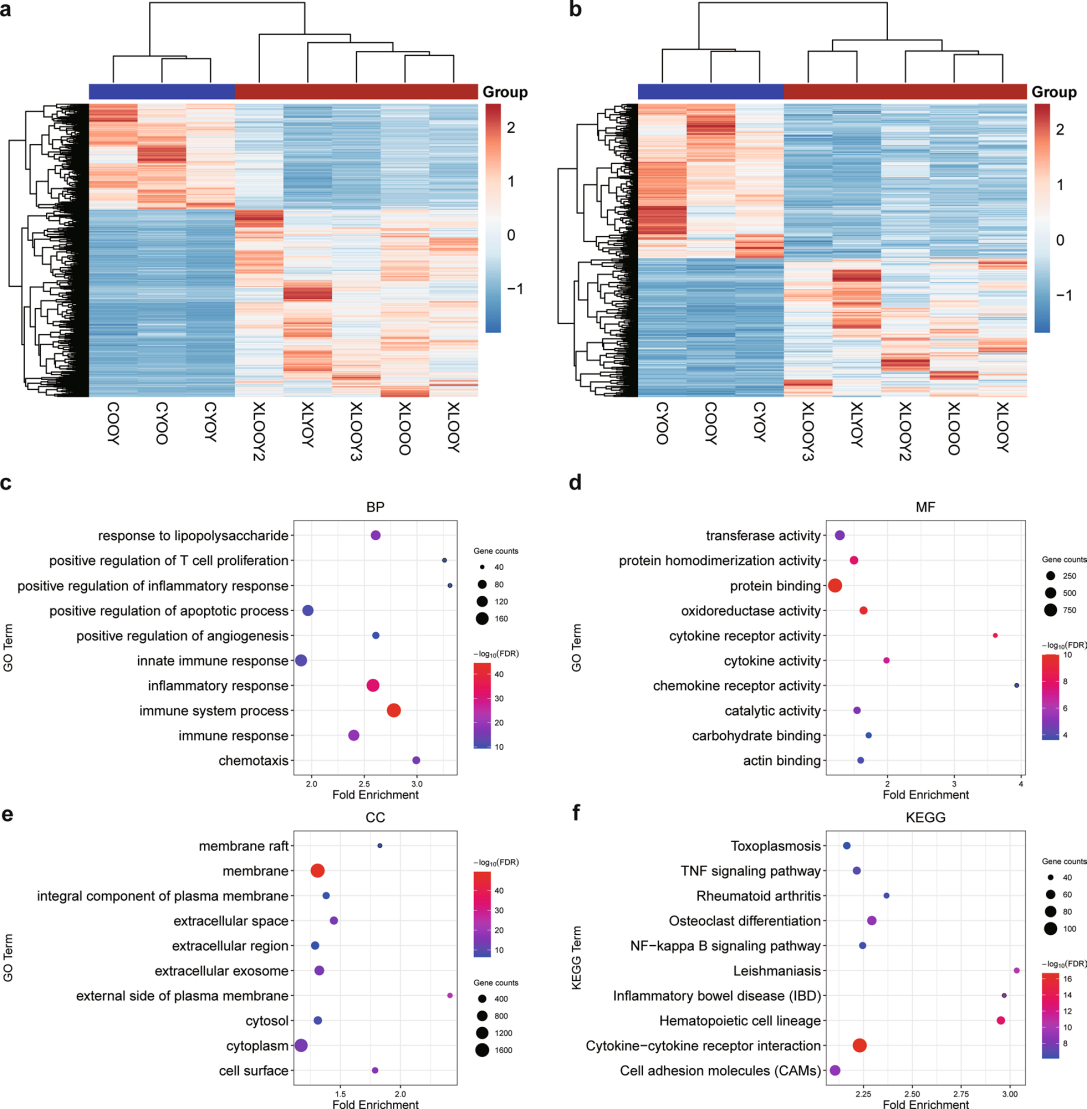
Functional enrichment analysis of differentially expressed mRNAs. **a**, **b** Hierarchical clustered expression analysis of differentially expressed mRNAs (**a**) and differentially expressed lncRNAs (**b**). Expression values for each gene are normalized across all samples based on the Z-score. **c**-**e** GO enrichment items of BP (**c**), MF (**d**) and CC (**e**). **f** KEGG analysis of differentially expressed mRNAs. BP, Biological process; CC, cellular component; GO, gene ontology; KEGG, Kyoto Encyclopedia of Genes and Genomes; MF, molecular function

Hierarchical clustering of DE mRNAs (Fig. 2a) and DE lncRNAs (Fig. 2b) indicated a clearcut discrimination between infected and control conditions, which suggested latent biological changes during infection. GO and KEGG analyses were performed to further investigate the crucial molecular events and processes. A series of classical liver injury pathways were significantly detected [35], including tumor necrosis factor (TNF)-related pathways, natural killer cell-related pathways, natural killer T (NKT) cell-related pathways, NF-kappa B-related pathways and interleukin-6 (IL6)-related pathways. Genes in those pathways were significantly enriched in DE gene sets (*P* < 0.001, Fisher’s exact test; detailed statistical information on 6 pathways is provided in Additional file 5: Table S3). Both innate and adaptive immune responses were enriched, suggesting comprehensive inflammatory activation due to parasite defense responses.

The significantly enriched biological process (BP) and molecular function (MF) GO terms involved immunological signatures, such as “innate immune response,” “positive regulation of T-cell proliferation,” “positive regulation of inflammatory response,” “chemokine receptor activity” and “cytokine activity” (Fig. 2c, d). Notably, most cellular component (CC) GO terms were related to membrane and extracellular interaction processes (Fig. 2e), implying that those genes were associated with excessive accumulation of extracellular matrix, as was reported in previous studies of liver damage in malaria [16].

Likewise, the significantly enriched KEGG pathways were related to immune processes (Fig. 2f). The uncovered pathways “cytokine−cytokine receptor interaction,” “cell adhesion molecules,” “NF−kappa B signaling pathway” and “TNF signaling pathway” indicated the links between cytokines and inflammatory responses, which have been reported in other *Plasmodium*–rodent systems and human malaria patients [36–38]. Intriguingly, the pathways “toxo–plasmosis” and “leishmaniasis”, which are involved in similar intracellular parasitic infections affecting internal organs, including the liver, were enriched. The autoimmune disease terms “rheumatoid arthritis” and “inflammatory bowel disease (IBD)” were unexpectedly enriched KEGG pathways, suggesting underlying cross-talk of immune responses between intracellular parasitic infections and autoimmunity due to the imbalance of pro-/anti-inflammatory responses [39, 40].

### GO term and KEGG pathway analysis of DE lncRNA-targeted genes highlighted metabolic disturbances

To explore the potential regulatory roles of DE lncRNAs, we predicted the targeted mRNAs of 493 DE lncRNAs by correlation of expression levels. A total of 1,599 mRNAs of 349 DE lncRNAs were identified as lncRNA-targeted mRNAs (correlation > 0.99 and adjusted *p* value < 0.01). Remarkably, metabolism-associated pathways were highlighted in the functional analysis of targeted mRNAs.

**Fig. 3:**
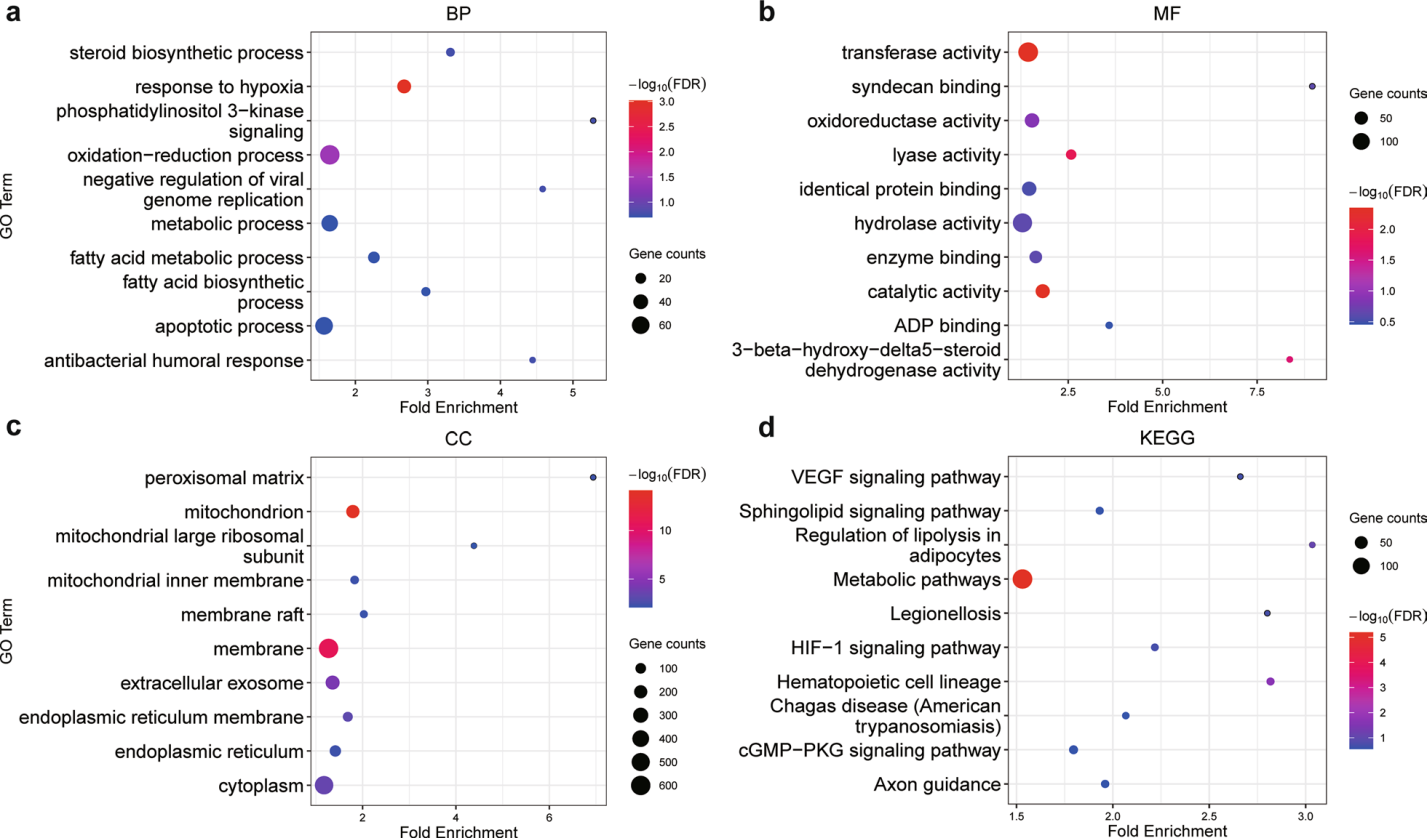
Functional enrichment analysis of differentially expressed lncRNA-targeted mRNAs. **a**, **b** and **c** GO enrichment items of BP (**a**), MF (**b**) and CC (**c**). **d** KEGG analysis of differentially expressed lncRNA-targeted mRNAs. BP, Biological process; MF, molecular function; CC, cellular component; KEGG, Kyoto Encyclopedia of Genes and Genomes

In BP enrichment terms, “fatty acid biosynthetic process,” “fatty acid metabolic process,” “oxidation−reduction process,” “steroid biosynthetic process” and “oxidoreductase activity” were the pivotal metabolic pathways associated with infection (Fig. 3a, b). CC and MF enrichment terms indicated the active status of the intracellular membrane and enzyme catalysis. These results suggested that lncRNA-targeted mRNAs predominantly contributed to metabolic flexibility during infection, such as in cellular lipid metabolism and oxidation reduction reactions (Fig. 3c).

Consistent with the GO enrichment results, KEGG analysis also revealed similar metabolism-related pathways, including “regulation of lipolysis in adipocytes” and “sphingolipid signaling pathway” (Fig. 3d). Additionally, hypoxia/ischemia response-associated pathways were detected. The term “HIF–1 signaling pathway,” which has been reported to be essential in the pathogenesis of severe malaria, highlights the hypoxic response because of pRBC sequestration and vessel occlusion [41]. The “cGMP−PKG signaling pathway” was found to be involved in protection against ischemia‒reperfusion injury [42], suggesting that similar multifocal damage occurred in the liver (Additional file 3: Fig. S2), which might be a consequence of clogging of microvessels.

### The host-parasite gene coexpression network indicated tissue remodeling-like changes

**Fig. 4:**
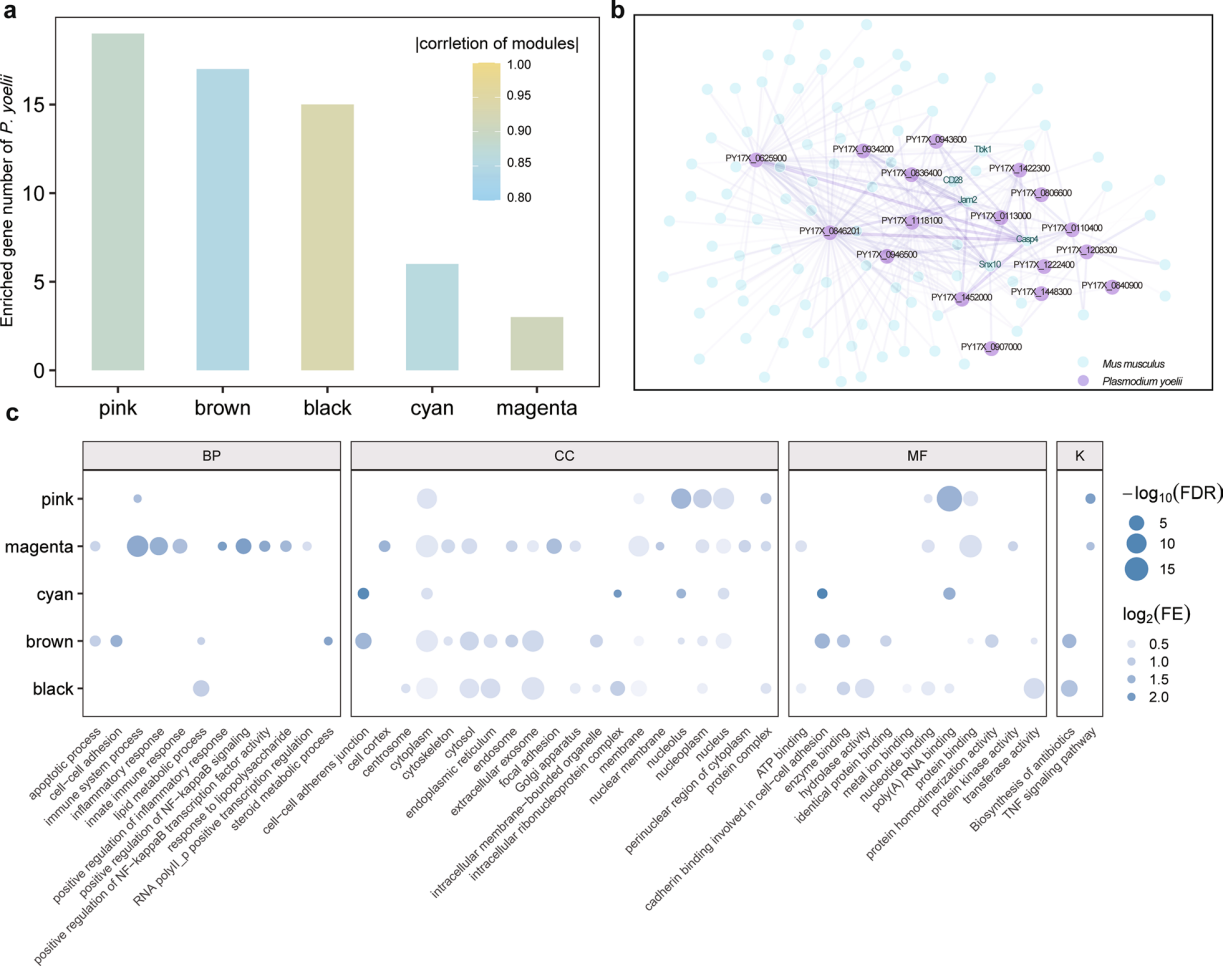
Coexpression network analysis of the dual RNA sequencing data of the host and *P. yoelii*. **a** Enriched genes of *P. yoelii* in modules (colors along* X*-axis) that are highly related to infection/control conditions. Low correlation coefficients of modules are shown in blue, and high correlation coefficients are shown in yellow (see scale at upper right). **b** Gene coexpression network of the pink module. The top 300 gene pairs ranked by their connectivity value are displayed. The purple dots represent genes of *P. yoelii*, and the green dots represent coexpressed host genes. The color shade and width of the lines between genes represent the connectivity. **c** Overlapping GO terms and KEGG pathways with FDR < 0.05 in five highly infection-related modules. The size of the dots represents -log_10_(FDR) and transparency represents log_2_(fold enrichment) of terms. FDR, False detection rate; FE, fold enrichment

During the infection process, the interaction between *P. yoelii* and the host results in large-scale changes in gene expression within both organisms. We subjected *Plasmodium*-containing liver samples to RNA-seq, with the aim to explore the simultaneous expression profiles of host and parasite, thus obtaining a novel perspective on host-parasite interactions. Through an artificial genome based on the reference genomes of both *M. musculus* and *P. yoelii*, we filtered 13,702 genes (386 parasite genes and 13,316 host genes) in dual RNA-seq. The PCA showed obvious differences between the infected and control groups. We then constructed a gene coexpression network, and five modules that were most highly related to infection were selected (Fig. 4a).

To minimize the background noise of the host-parasite cotranscriptome, we considered only *P. yoelii* genes in the top 1000 soft-connectivity of each module to be potential host-influencing genes. The five selected infection-related modules contained a total of 60 eligible genes (Fig. 4a). In the module “pink,” which contained the most parasite genes, the most highly coexpressed host genes included the endosome homeostasis-related gene *SNX10* [43], the apoptosis/inflammation-related gene *Casp4* [44], the liver fibrosis-related gene *Jam2* [45], the ischemia/reperfusion regulating gene *Tbk1* [46] and the lymphocyte activation-related gene *Cd28* [47] (Fig. 4b), indicating elevated inflammation levels and activated immune responses that were consistent with the previous results of functional enrichment (Fig. 2c, f).

Further functional enrichment analyses of all highly coexpressed host genes revealed 67 overlapping terms that were detected in at least three modules (Fig. 4c; Additional file 6: Table S4). A predominant portion of these terms was associated with dynamic changes in the cellular transport system, including “cytoskeleton,” “cell cortex,” “endocytosis,” “Golgi apparatus,” “endosome,” “early endosome,” “intracellular membrane-bounded organelle,” “mitochondrial outer membrane,” “protein transport” and “extracellular exosome”, reflecting a complex intercellular and extracellular interaction network. Moreover, terms related to cell‒cell connections were also significantly enriched, such as “cadherin binding involved in “cell‒cell adhesion,” “cell‒cell adhesion,”, “focal adhesion” and “cell‒cell adherens junction”, which highlighted the altered tissue structure and histological environment during liver damage. The terms “angiogenesis”, “regulation of cell proliferation” and “apoptotic process” demonstrated that the tissue remodeling processes were similar to those of chronic liver diseases [48], which might be the adaptive repair of tissue injury.

Immunity-related genes were enriched in “positive regulation of inflammatory response,” “innate immune response,” “response to cytokine,” “positive regulation of NF-kappa B transcription factor activity,” “positive regulation of I-kappa B kinase/NF-kappa B signaling” and “TNF signaling” pathways. Specifically, the metabolic term “biosynthesis of antibiotics” was uncovered in the KEGG pathway, which might be related to microbial translocation occurring in the early stage of liver diseases associated with *Plasmodium* burden [49].

### Conserved and nonconserved coexpressed genes of* P. yoelii*

The 60 hub genes of *P. yoelii* identified in the coexpression network were mainly composed of energy biogenesis-related genes, FAM genes, Ras family genes and transcription/translation-related genes. We next concentrated on the homologous genes of other *Plasmodium* species to enhance our understanding of these protein-coding genes and thus shed light on the pathogenesis and virulence of *Plasmodium* spp. The *Plasmodium* genomes of the human parasites *P. falciparum* [50], *P. malariae* [51], *P. ovale* [51], *P. vivax* [52] and *P. knowlesi* [53] as well as the rodent parasites *P. berghei* [54] and *P. chabaudi* [54] were included for homology identification. Taking an e-value < 1 × 10^–10^ as the threshold, only *P. yoelii* proteins with > 90% overlapping sequences with other *Plasmodium* proteins and overlapping sequences accounting for > 90% of the two *Plasmodium* protein sequences were kept for downstream analysis (Additional file 7: Table S5).

**Fig. 5:**
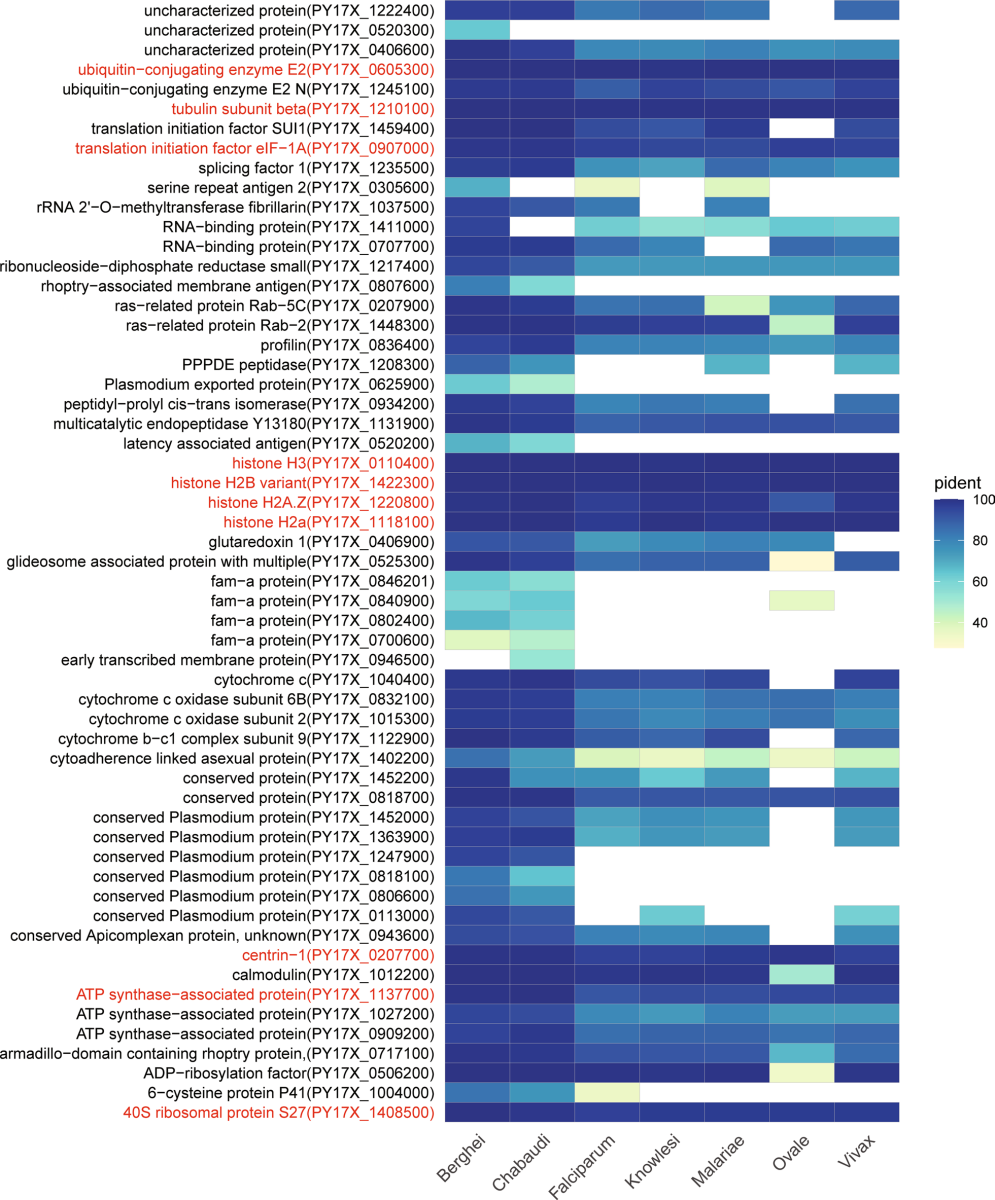
*Plasmodium* homolog of the hub genes of *P. yoelii* in the parasite-host coexpression network. Protein identity is distinguished by different colors, as indicated in the legend. Ten conserved *P. yoelii* genes among seven *Plasmodium* spp. are highlighted in red. IL, Interleukin; NF-κB nuclear factor kappa B; NKT, natural killer T; TNF, tumor necrosis factor

Of these 60 hub genes, three failed homology screening, which included genes encoding an uncharacterized protein, a *Plasmodium* exported protein of unknown function and an S-antigen. S-antigen is assumed to contribute to the extensive malaria antigenic diversity of the asexual blood stage of the parasite, which is considered useful in the stereotypic analysis of natural parasite populations [55]. As expected, more conserved genes of *P. yoelii* were found in rodent parasites *P. berghei* and *P. chabaudi* than in human parasites. Ten *P. yoelii* genes were identified as conserved among seven *Plasmodium* spp. (Fig. 5), including four histone genes, the 40S ribosomal protein S27 gene, the translation initiation factor eIF-1A gene, the tubulin subunit beta gene, the centrin-1 gene and the ubiquitin-conjugating enzyme E2 gene. Tubulin and centrin are cytoskeleton proteins with key roles in cell division, such as merozoite biogenesis [56, 57]. Ubiquitin-conjugating enzyme E2 and the less conserved ubiquitin-conjugating enzyme E2 N are involved in ATP-dependent ubiquitination, which has been acknowledged to be involved in the growth and adaptation of apicomplexan parasites [58].

### Discussion

Jaundice, hepatocyte dysfunction and hepatic encephalopathy are common severe manifestations in patients with malaria [19, 59–61] and are diseases believed to be associated with complex complications. However, the direct roles of the liver in the blood stage of the parasite remain unclear. In our study, using a *P. yoelii*-rodent model system, we demonstrated the molecular characteristics of the infected mouse liver during the blood stage of *P. yoelii* infection, when activated immune responses, disrupted lipid metabolism, extensive hypoxia/ischemia stress and accompanying tissue remodeling were significant biological events.

Infection-specific perturbations in lipid metabolism have been demonstrated in malaria patients, and these could contribute to poor clinical outcomes [62–64]. The intertwined metabolic pathways of the host and parasite are preferentially studied integrally, but this approach leads to the loss of detailed information on specially involved organs. Our results show that the lipid metabolism processes of hepatocytes were also disturbed. Whether there exists a correlation between aberrant liver metabolism and disordered metabolomics remains to be clarified.

Coinfection with parasites and bacteria is a noteworthy clinical issue in malaria-endemic regions and is associated with concomitant sepsis and poor prognosis [65–67]. Approximately 6% of children with severe *P. falciparum* malaria were observed to suffer from invasive bacterial diseases, such as nontyphoidal *Salmonella* [65, 68, 69]. In our study, the bacterial immune response was detected in DE mRNA and host-parasite coexpression functional analysis, suggesting that a potential concomitant bacterial invasion occurred in *Plasmodium* infection. The liver has a unique vascular system within the gastrointestinal tract, as the majority of the liver's blood supply comes from the gut via the portal vein. The gastrointestinal tract microenvironment has been demonstrated to be the target of *Plasmodium* parasites [70]. A malaria infection can cause disruption of the vascular and epithelial barriers in the gut, leading to dysfunction of gut microbiota homeostasis. Long-term liver damage was found to be associated with altered gut microbes in a malaria mouse model [71], while increased abundances of some probiotics were found to decrease the parasite burden [72]. The resolution of this three-way crosstalk between host-plasmodium-microbiota may help to elucidate immune responses, anti-parasitic responses and regulatory roles of the gut microbiota, exploiting the gut microbiota as novel targets for antimalarial treatment.

The dual RNA-seq approach allows parallel analysis of the host and pathogen transcriptomes, providing profound insights into a comprehensive understanding of host‒pathogen interactions. In our study, hHost genes highly coexpressed with *P. yoelii* genes were mainly focused on processes of tissue remodeling, which was similar to the structural reconstruction that is observed in chronic liver diseases [73]. We hypothesized that sinusoid disorder might worsen hepatocyte hypoxia and disrupt portal circulation, as occurs in other peripheral organs [74], which leads to further liver dysfunction and provides shelter for parasites. This immune escape might be an explanation for the higher incidence of complications in patients with malarial hepatitis, but more experiments are needed to prove this hypothesis.

Conserved genes reflect common responses, while nonconserved genes indicate unique changes in species. In our study, three out of 60 *P. yoelii* genes failed homology screening, including the S-antigen gene. Despite the unknown function and polymorphism of the S-antigen system among strains, this system is capable of eliciting antibodies with neutralizing properties [46], which may warrant revisiting in future vaccine studies of *P. yoelii*. Conserved genes related to cytoskeleton ubiquitination are coexpressed with host genes in the parasite-host network. Several microtubule inhibitors are reported to block the growth and development of malaria parasites at certain concentrations [75, 76]. The presented selective toxicity is partly due to different tubulin affinities, rendering microtubule inhibitors with different species affinities a promising anti-malaria approach. The type and chain length of ubiquitination further determines whether the protein is assigned to play further roles in multicellular processes, such as transcription. Ubiquitination of the E3 ligase NEDD4 has been reported to result in downregulation of autophagy-related genes in exoerythrocytic forms (EEFs) during the liver stage of the parasite in the *Plasmodium berghei* ANKA model, potentially promoting survival of and completion of hepatocyte infection by EEFs [77]. Due to the significant structural conservation of several ubiquitination genes, drugs targeting the ubiquitin system could potentially be developed into antimalarial agents with promise for overcoming resistance [78–81].

The *P. chabaudi*–rodent model is another malaria mouse model that has been used in recent years to study liver pathology, with abnormal metabolism and activated immune responses reported using this model. Hepatic cytochrome 450 (CYP) activity has been reported to be regulated in the *P. chabaudi*-mouse model, which may further interfere with drug metabolism [82]. We noted that in our study, in addition to identifying both *Cyp2a5* upregulation and *Cyp1a2* downregulation, we found that the gene expression levels of several genes of the CYP family 2, subgroup family c (*Cyp2c23*, *Cyp2c29*, *Cyp2c40*, *Cyp2c50*, *Cyp2c55*, *Cyp2c67*, *Cyp2c78*, *Cyp2c69*, *Cyp2c70*) were also significantly decreased under infection conditions. Similar to our enriched immune pathways, activated immune responses, such as pro-inflammatory IL-1α, anti-inflammatory factors IL-22, TNF-α and hepatic hemozoin levels, were demonstrated in previous studies to be involved in the development of liver pathology in the *P. chabaudi*-mouse model [14, 21, 22, 83]. Further transcriptomic studies performed on *P. chabaudi*-mouse models showed that innate immune responses and adaptive immune responses play distinct roles in the acute and recrudescence stages of the parasite [84, 85]. Considering the differences between *P. chabaudi* and *P. yoelii* [86], detailed liver changes need to be studied and compared to different clinical liver damage manifestations in human malaria.

Taken together, our study presents a systematic overview of biological changes in the *P. yoelii-*infected mouse liver during the blood stage of the parasite, in which activated immune responses, aberrant metabolism processes and tissue remodeling changes were the dominant molecular characteristics. However, the small amount of *P. yoelii* in the liver and the restricted sequencing depth limited our research to partial parasite genes in the coexpression network analysis; hence, multi-organ studies with deeper depth are required for further insight into host-parasite interactions. We expect that our findings will advance current understanding of malaria complications and facilitate the design of novel interventions to improve the clinical outcomes of severe malaria.

## Conclusions

Activated immune responses, aberrant metabolic processes and tissue remodeling changes were the dominant molecular characteristics in the livers of *P. yoelii*-infected mice during the blood stage of the parasite. Future work will require the use of experimental approaches to demonstrate these changes as well as the roles of the liver in malaria.

## Supplementary Information

**Additional file 1: Table S1.** Primers of the top 10 mRNAs and top 10 lncRNAs used for RT‒qPCR validation.

**Additional file 2: Figure S1.** Blood smears of five infected mice on day 5 post-infection.

**Additional file 3: Figure S2.** Parasitemia and histopathology of mouse liver.** A** HE staining of control mouse liver.** B**,** C**,** D** HE staining of *P. yoelii*-infected mice.

**Additional file 4: Table S2.** Detailed information on RNA-seq.

**Additional file 5: Table S3.** GO terms related to liver injury and Fisher's exact test of term enrichment.

**Additional file 6: Table S4.** Overlapping GO and KEGG terms of coexpression modules.

**Additional file 7: Table S5.** Homologous genes among *Plasmodium* spp.

## Data Availability

The code for this study can be found in the GitHub repository (https://github.com/yukkikou/Mus_Pyoelii_2021). The clean data are available at NCBI under accession number PRJNA782413 (http://www.ncbi.nlm.nih.gov/bioproject/782413).

## Abbreviations

BP: Biological process
CC: Cellular component
DE: Differentially expressed
EEFs: Exoerythrocytic forms
GO: Gene ontology
KEGG: Kyoto Encyclopedia of Genes and Genomes
lncRNA: Long noncoding RNA
MF: Molecular function
mRNA: Messenger RNA
PCA: Principal component analysis
pRBCs: *Plasmodium*-infected red blood cells
